# A Cognitive Neurodynamic Approach to Prediction of Students’ Adaptation to College: An Ex-Post Facto Study

**DOI:** 10.29252/NIRP.BCN.9.3.217

**Published:** 2018

**Authors:** Seyed Alireza Derakhshanrad, Emily Piven

**Affiliations:** 1. Department of Occupational Therapy, School of Rehabilitation Sciences, Shiraz University of Medical Sciences, Shiraz, Iran.; 2. Rehabilitation Sciences Research Center, Shiraz University of Medical Sciences, Shiraz, Iran.; 3. Health Matters First of Florida, Inc., Oakland, Florida, United States.

**Keywords:** Circular causality, Cognitive neuroscience, Intention, Meaning, Mental process, Perception

## Abstract

**Introduction:**

Campus life tends to make social and academic demands on college students. To cope with these demands, students are required to use their neurocognitive skills of problem-solving and planning intentional actions that target towards adaptation to college. This paper presents an illuminating perspective that would inform understanding of a new approach to cognitive neuroscience. The linkage between cognition and adaptation was sought in the context of a cognitive neurodynamic approach proposed by the Intention, Meaning, and Perception (IMP) model of neuro-occupation.

**Methods:**

An ex post facto study was conducted on a convenience sample of 187 college students in Shiraz, Iran. A brief questionnaire was developed to screen participants for diversity of cognitive neurodynamic processing capacity and three standardized questionnaires were used to gather data about college adaptation manifestations. The partial correlation, 1-way, and 2-way ANOVA tests were used to analyze the data.

**Results:**

The partial correlation test showed large, positive correlation (r≥0.7, P<0.001) between elements of the cognitive neurodynamic process, denoting that the interrelated connections among intention, meaning, and perception were governed by feedback loops. One-way ANOVA test revealed that students with diverse cognitive neurodynamic processing capacity had a variety of college adaptation manifestations. Two-way ANOVA showed a statistically significant main effect for neurodynamic processing capacity (F_2, 178_=8.1, P<0.001).

**Conclusion:**

College adaptation could have been established by the cognitive neurodynamic process proposed by the IMP model. Therefore, it is advisable for faculty, mental health practitioners, and counselors who work with students at universities to understand this process and address students’ maladaptation to campus life.

## Highlights

- The theoretical framework of cognitive neurodynamic approach introduced in this study provides an understanding of the linkage between neurocognitive skills and students’ college adaptation.
- Students’ cognitive neurodynamical process of intention, meaning, and perception generates the impetus for successful adaptation to campus life.

## Plain Language Summary

This study introduced and examined a useful theoretical framework, the cognitive neurodynamic approach, which explains the linkage between neurocognitive skills and students’ adaptation to college. The study is importantbecause of few empirical studies in this area, leading to confusion about the association between neurocognitive skills and adaptation to college life. Introducing the cognitive neurodynamic approach, this study helps the reader understand the relationship between cognition and college adaptation. Moreover, this research can help influence college policy makers as well as student counselors to pay more attention to how students cope with educational demands through understanding planning their intentional actions to engage in the problem-solving process. The study findings illustrate how the cognitive neurodynamic approach demonstrates the linkage between neurocognitive skills and college adaptation manifestations, thus enhancing the ability of counselors and professors to easily and quickly screen the students who may have difficulty in adapting to campus life. The results suggest that cognitive neurodynamic process may be a significant predictor of college adaptation. It may also indicate that students’ motivation, identification of meaningful experiences, and perception of themselves as individuals who are efficacious and satisfied with the outcomes of their academic performance, may be embedded and best expressed in the cognitive neurodynamic approach.

## 1. Introduction

Campus life tends to make plenty of demands on college students. For this reason, adaptation to college is vital for students’ mental and physical health (Zea, Jarama, & Bianchi, 1995). To cope with educational demands, students are required to use their neurocognitive skills for problem- solving and planning intentional actions targeted towards college adaptation (Cousins, Servaty-Seib, & Lockman, 2015; Sasaki & Yamasaki, 2007). Successful adaptation to demands of the college environment enables best academic performance by adopting productive educational habits (Credé & Niehorster, 2012). It is now generally recognized that failing to adapt to college may be detrimental to health and academic performance, while succeeding in adaptation to college may lead to academic achievement (Baker, 2004; Consolvo, 2002; Katz & Somers, 2015; McKenzie & Schweitzer, 2001). With this end in view, it should be noted that college adaptation focuses mainly on the construct of “students’ adjustment to college” (p. 134), rather than “adjustment of college student” (p. 134). The former is concerned with the students’ ability to effectively adapt to the numerous educational challenges encountered in college context, while the latter is referred to coping with challenges that are not necessarily tied to college context or university environment, but may already be present due to a traumatic event or other events in a student’s personal life (Credé & Niehorster, 2012).

Studies have revealed that neurocognitive skills tend to be a possible predictor of students’ adaptation (Credé & Niehorster, 2012; Zea et al., 1995). A cognitive approach known as Cognitive Appraisal Coping (CAC) model has been the common theoretical framework that underpins research in this area (Cousins et al., 2015). In this model, cognition is referred to as a constantly changing, process-oriented approach that leads a person to adapt through managing external and or internal demands (Lazarus & Folkman, 1984).

The CAC model, commonly used as an underpinning theoretical framework for study of adaptation of college student, might be of little interest for study of students’ adaptation to college for this study. For example, one study guided by CAC model examined how college students made adaptation following stressful life events (Schnider, Elhai, & Gray, 2007). A second study examined the adaptation of college students facing the challenge of coping with the death of someone they considered important in their lives (Cousins et al., 2015). The existing empirical literature provides limited information about the theoretical frameworks that underpin the association between neurocognitive skill and adaptation to college. Therefore, it would seem that further investigations are needed, in order to provide a useful theoretical framework to understand this linkage.

### 1.1. Neurocognitive skills and college adaptation

It is said that the neurocognitive skill that reinforces college adaptation is an intellectual or a mental process involving motivation, compatibility, and satisfaction with the academic performance and environment. In other words, neurocognitive skills that addresses the students’ college adaptation comprises three main elements, including determination, assimilation of meaning through academic performance, and perception of self-efficacy regarding such performance (Baker, 2004; Rienties, Beausaert, Grohnert, Niemantsverdriet, & Kommers, 2012). Furthermore, college adaptation manifestations are associated with particular behaviors, including (a) enthusiasm for being in college and doing college work; (b) expressive effort to do academic tasks; and (c) effective or successful effort expended (Alt & Itzkovich, 2016).

With this end in view, this paper presents an illuminating perspective that would inform understanding of the linkage between neurocognitive skill and college adaptation using a cognitive neurodynamic approach proposed by the Intention, Meaning, and Perception (IMP) model of neuro-occupation. This approach, outlined by Freeman (1999, 2000a) and developed by Lazzarini (2004), named the neurocognitive skill development as the cognitive process of circular casuality.

### 1.2. Cognitive process of circular causality

As has been presumed, the IMP model may be suitable for exploring the complexity of the college adaptation phenomenon, by taking cognitive process of circular casuality into account. Cognitive process of circular causality is an intellectual or mental process that is composed of three levels: intention, meaning, and perception. Intention is defined as the state of brain readiness that motivates one to fulfil his or her needs and desires. Meaning refers to the ability to assimilate meaning following one’s performance. Perception is known as one’s attitude, feeling and beliefs that form one’s self-perceived concept (Derakhshanrad et al., 2017a).

The IMP model clarifies “a fresh approach to explaining adaptation and stimulates a better appreciation of the uniqueness of the individual” (Haltiwanger, Lazzarini, & Nazeran, 2007). Presuming that adaptation is the desired outcome of the cognitive process of circular causality, the IMP model describes the process as the result of interdependent relationships that develop intellectually between one’s intention, meaning, and perception levels. This cognitive neurodynamic process is seen as circular, whereby plenty of feedback loops form. That is, the result of the circular process is the intentions and meanings that lead to establishment of new perception(s). Hence, the latest perception(s) determine the upcoming intentions and meanings in a circular feedback process (Freeman, 1999). The effect of this cognitive neurodynamic process is to create informing patterns for individuals. “These informing patterns provide the windows by which we look into the world, and by which we see ourselves peering into our own windows” to develop insight (Freeman, 2005). In other words, cognitive neurodynamical circular causality process of intention, meaning and perception enables humans to be adaptive agents, who successfully cope with environmental constraints (Freeman, 1999; 2003).

This study is a preliminary attempt to objectively quantify the cognitive process of circular causality. Therefore, the purpose is to test the IMP model by examining the correlations between cognitive neurodynamic process elements of intention-meaning-perception, as well as by comparing college adaptation manifestations among students with diverse cognitive neurodynamic processes.

### 1.3. Hypotheses

The premises of IMP model would suggest two key hypotheses to be formulated. First, we could expect to find statistically significant correlations between neurodynamic elements of intention, meaning, and perception. Second, we could propose that students with diverse cognitive neurodynamic process have different college adaptation manifestations, as revealed by their different motives, and different meaningful and self-perceived experiences. Provision of support for these hypotheses would be desirable for several reasons. First, understanding the trend of correlations between a student’s intention, meaning, and perception neurodynamic process may help reveal the foundations of adaption to college phenomenon. Second, outcomes of this study might potentially be used to help policy makers at colleges and universities provide students with college-based support services with future higher level research. In addition, educational mental health practitioners and professors can plan attractive, motivating educational demands that may potentially promote college adaptation through facilitating the cognitive process of circular causality.

## 2. Methods

### 2.1. Study design

To test the two research hypotheses, an ex post facto study was conducted. Ex post facto or causal-comparative research is a retrospective type of descriptive research to analyze differences between groups non-experimentally (Carter, Lubinsky, & Domholdt, 2011).

### 2.2. Settings and participants

The convenience sample included all of the second-year, junior, and senior undergraduate Occupational Therapy (OT), Physical Therapy (PT), and Speech Therapy (ST) Persian students studying at Shiraz School of Rehabilitation Sciences, Iran. All students spoke Farsi as their native language. Having been selected after passing the national entrance exam held once annually all over the country (Iran), the students of Shiraz School of Rehabilitation Sciences were a convenience sample that could be considered a representative sample of an Iranian student population, as they were from various ethnic and economic family groups.

### 2.3. Study instruments

The research materials consisted of a brief questionnaire used to screen participants for their cognitive neurodynamic processing capacity, called the Brief Questionnaire of Cognitive Neurodynamic Processing Capacity (BQCNPS). In addition, three standardized questionnaires were available in Persian, to gather data about college adaptation manifestations. College adaptation was defined earlier as the behavioral manifestations of students, expressed as enthusiasm for doing college work, obtaining meaning from their studies, and their effective academic efforts (Alt & Itzkovich, 2016). Thus, three questionnaires were used to gather data about college adaptation manifestations: The Hermans achievement motivation questionnaire, the meaning in life questionnaire, and the academic self-efficacy questionnaire. These questionnaires have been applied widely in Iranian studies and on college students. The questionnaires were developed through forward and backward translation of the original scales into Persian with questions adapted to the Iranian culture.

#### 2.3.1. Hermans Achievement Motivation Questionnaire

Since the Hermans achievement motivation questionnaire measures the construct of achievement motivation of students (Hermans, 1970), it was considered appropriate for this study. This questionnaire contained 29 incomplete sentences that should be completed by examinee through choosing a preferable choice from a pool of four options provided. Each item is scored between 1 and 4 such that the total scores range from 29 to 116; the higher scores identified the student with higher enthusiasm for doing academic tasks. The questionnaire was standardized on 1073 (560 females and 513 males) high school students of city of Saveh, Iran. The validity and reliability of the questionnaire were approved by performing the construct validity procedure and the Cronbach α of 0.803 (Hoomon & Asgari, 2001).

#### 2.3.2. Meaning in Life Questionnaire

The meaning in life questionnaire was chosen for the present study because it measures the presence of and search for meaning in life (Steger, Frazier, Oishi, & Kaler, 2006). It is used to measure whether the student assimilated his or her current life meaningfulness. This 10-item questionnaire rated each item on a 7-point Likert-type scale from strongly disagree to strongly agree. The scores ranged from 10 to 70, whereby the higher scores meant the student was likely to assimilate campus life meaningfully. The applicability of this questionnaire in Iranian students was confirmed through a study on 296 college students with 17–29 years of age that examined confirmatory factor analysis, as well as discriminative and constructs validity of the questionnaire (Mesrabadi, Jafariyan, & Ostovar, 2013).

#### 2.3.3. Academic Self-Efficacy Questionnaire

The academic self-efficacy questionnaire was chosen for the present study because it measured the student’s belief in his or her ability to meet the educational demands of the coursework (Davidson, Beck, & Silver, 1999). Rated on a 5-point Likert-type scale from strongly disagree to strongly agree, this was a 6-item questionnaire. The scores range from 6 to 30, where higher scores revealed highly efficacious students that more satisfied with their academic performance. Psychometric properties were examined from a sample of 200 Iranian nursing college students of 19–36 years of age. The validity and reliability of the questionnaire were approved by the content validity and the Cronbach α of 0.81 (Rafii, Sarami Rasouli, Najafi Ghezeljeh, & Haghani, 2014).

#### 2.3.4. Brief Questionnaire of Cognitive Neurodynamic Processing Capacity

To explore the diversity in students’ cognitive neurodynamical circular causality process of intention, meaning and perception, a brief questionnaire, called the Brief Questionnaire of Cognitive Neurodynamic Processing Capacity (BQCNPS), was developed based on intention, meaning, and perception definitions proposed by Derakhshanrad et al. (2017a). Each item was designed to respectively examine intention, meaning, and perception components of circular causality cognitive process. The items were as follows: 1. Choose a number between 0 and 10 to show your mental readiness for doing academic tasks; 2. Choose a number between 0 and 10 to demonstrate how much you assimilate all your college educational demands; and 3. Choose a number between 0 and 10 to demonstrate your belief in your ability to meet the educational demands. Overall, higher scores were hypothesized to be associated with more effective cognitive neurodynamic processing capacity.

### 2.4. Data analysis

Verifying the assumption of normality and controlling for age, the partial correlation test was used to test the first research hypothesis. Partial correlation is similar to the Pearson correlation, but it allows the researcher to control for additional variables (Pallant, 2013). Accordingly, using the scores obtained from each item from the BQCNPS, the partial correlation was run to test whether there were statistically significant correlations between the three elements of the cognitive neurodynamical circular causality process of intention, meaning and perception. To test the second hypothesis, 1-way ANOVA test with post-hoc comparisons using the LSD test was selected to compare the mean scores of Hermans achievement motivation, meaning in life, and academic self-efficacy questionnaires between the students, who had diverse cognitive neurodynamic processing capacity as indicated by the BQCNPS. SPSS version 23 was used to analyze the data. To choose another clustering method that showed the utility of the BQCNPS, an additional 1-way ANOVA was performed to compare total scores obtained from three standardized questionnaires among students with diverse cognitive neurodynamic processing capacity. A 2-way between-groups analysis of variance was also conducted to explore the impact of the major (OT, PT, ST) and cognitive neurodynamic processing capacity on college adaptation. Significance was set at P<0.05 for all tests.

### 2.5. Study Procedure

This research was approved by Research Ethics Committee of Shiraz University of Medical Sciences, Iran. Informed consent was obtained from all participants. The instruction to students and administration of the research materials were done in classrooms. They were also provided with enough time to complete the research instruments. Sampling was conducted over two months from the beginning of November to the middle of December, 2016.

## 3. Results

The total number of second-year, junior, and senior OT, PT, and ST students studying at Shiraz School of Rehabilitation Sciences was 191, of whom 187 (97.9%) students (54 male and 133 female) participated creating a convenience sample for the study. The mean age of participants was 21.3 year (SD=1.7) with minimum and maximum of 18 and 34 years, respectively. Of those who participated in the study, 73 were OT, 62 were PT, and 52 were ST students. In addition, 53 students were second-year, 64 were junior, and 70 were senior students.

**Figure 1:**
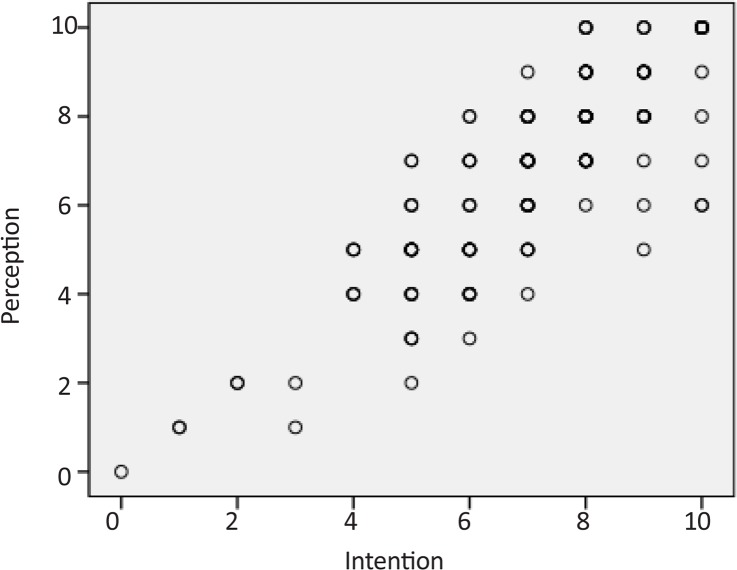
Scatterplot of correlation between intention and perception

**Figure 2:**
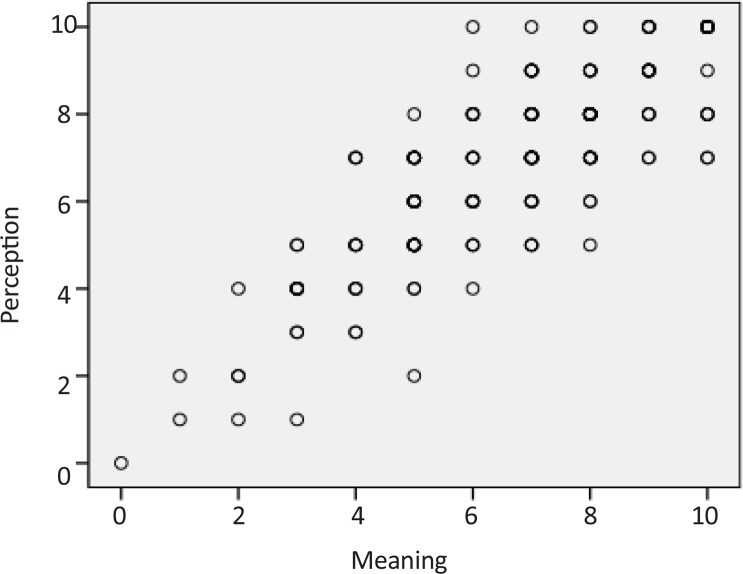
Scatterplot of correlation between meaning and perception

**Figure 3:**
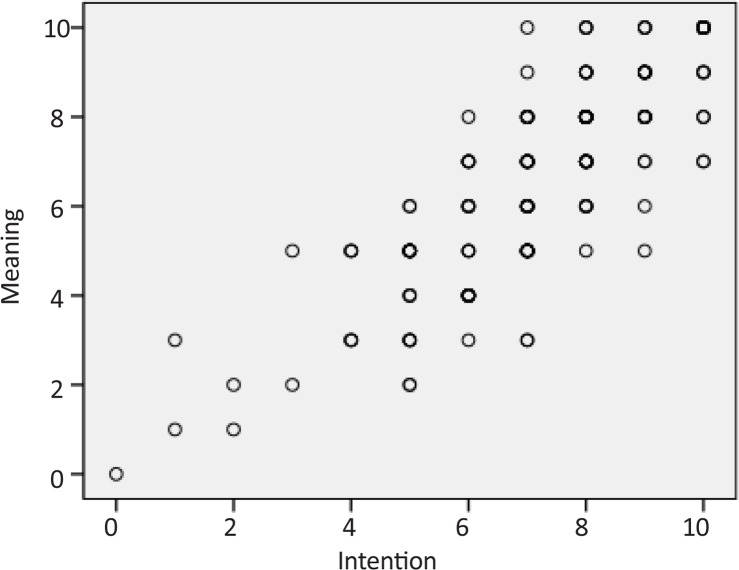
Scatterplot of correlation between intention and meaning

There were no statistically significant differences between mean±SD ages of OT (21.4±1.9), PT (21.3±1.3), and ST (21.2±1.3) students (P=0.637). Using the score from each item of the BQCNPS to run the partial correlation test revealed large, positive correlations among the three elements of cognitive process of circular causality, denoting that the interrelated connections among intention, meaning, and perception were governed by feedback loops. Table 1 shows the results of this test. Figures 1, 2, and 3 provide the scatterplots of correlations between intention, meaning, and perception variables to indicate the nature of the relationships between them.

**Table 1:** Partial correlations between intention, meaning, and perception

**Control Variable (Age)**	**1**	**2**	**3**
1. Intention	-		
2. Meaning	0.70*	-	
3. Perception	0.71*	0.78*	-

* P<0.001 (2-tailed).

Prior to testing the second hypothesis, participants were divided into three groups according to their scores on the BQCNPS. The first group of students who scored from 0 to 15 on the test was presumed to have low cognitive neurodynamic processing capacity. Then, assumed to have moderate cognitive neurodynamic processing capacity, the second group of students were composed of those who scored from 16 to 24 on the test. Finally, the group known as students with high cognitive neurodynamic processing capacity, were composed of those who scored from 25 to 30 on the test. Then, 1-way between-groups ANOVA was performed to explore whether three groups of students with low, moderate, and high cognitive neurodynamic processing capacity had different college adaptation manifestations, as measured by the Hermans achievement motivation, meaning in life, and academic self-efficacy questionnaires. Table 2 shows the results of 1-way ANOVA tests.

**Table 2:** Results of ANOVA test

**Questionnaires**	**Neurodynamic Processing Capacity Groups**			**F**	**Sig. (2-tailed)**

	**Low Mean (SD)**	**Moderate Mean (SD)**	**High Mean (SD)**		
Hermans achievement motivation	83.2(8.1)	84.5(7.6)	86.7(9.5)	2.4	0.044
Meaning in life	52.1(10.8)	55.7(8.1)	58.5(10.01)	5.3	0.006
Academic self-efficacy	18.1(3.7)	19.8(3.4)	21.3(5.01)	7.6	0.001

Post-hoc comparisons using the LSD test indicated the statistically significant differences in mean scores between three groups of cognitive neurodynamic processing capacity, while analyzing the results obtained from each questionnaire. The effect size, calculated using eta squared, was 0.03 for Hermans achievement motivation questionnaire, 0.06 for meaning in life questionnaire, and 0.08 for academic self-efficacy questionnaire. As evident from the effect sizes, the differences in the mean scores between the groups were small. However, the statistically significant results were of particular importance because the sample (187 in this case) was not too large to be responsible for the differences to become statistically significant (Pallant, 2013). Still, these findings need further support to be confirmed.

To further analyze the utility of the BQCNPS, an additional 1-way ANOVA was conducted to compare total scores obtained from three standardized questionnaires among three groups of cognitive neurodynamic processing capacity. There was a statistically significant difference at the P<0.05 level in college adaptation manifestation scores for the three neurodynamic processing capacity groups (F _2, 184_ =8.4, P<0.001). The LSD test used for post-hoc comparisons of mean score on college adaptation for the low processing capacity group (Mean=153.6, SD=15.7) was significantly different from the moderate processing capacity group (Mean=160.1, SD=13.8) (P=0.17), and the high processing capacity group (Mean=166.6, SD=16.9) (P<0.001). The moderate processing capacity group did also differ significantly from high processing capacity group (P=0.18).

A 2-way ANOVA was conducted to explore the impact of the chosen major (OT, ST, PT) and cognitive neurodynamic processing capacity (low, moderate, high) on college adaptation, as measured by three standardized questionnaires. There was a statistically significant main effect for cognitive neurodynamic processing capacity (F _2, 178_ =8.1, P<0.001). The main effect for the chosen major (F _2, 178_ =0.9, P=0.4) and the interaction effect of the major and cognitive neurodynamic processing capacity (F _4, 178_ =0.54, P=0.7) did not reach statistical significance.

## 4. Discussion

In this study, we investigated how the IMP model explained the linkage between neurocognitive skill and college adaptation manifestations, thus enhancing our knowledge to easily and quickly screen the students who may have difficulty adapting to campus life. The survey reported on this study has revealed two key findings, as described below.

First, as measured by the brief questionnaire, elements of circular causality cognitive process had large, positive correlations with each other to shape the students’ cognitive neurodynamic processing capacity. According to the results, these elements were highly associated with each other because they describe circular causality feedback loops, whereby each element has an influence on the other to produce adaptation to college in this research. Although its confirmation needs more research, the high association was not an unexpected finding because the circular causality cognitive process “is not simply one of input-throughput-output (as in linear causality), but one of circular causality through the interrelated processes of intention, meaning, and perception” (Champagne, Ryan, Saccomando, & Lazzarini, 2007).

Second, the results showed that when a student’s score on BQCNPS was high, there would be a good possibility that he or she had more effective cognitive neurodynamic processing capacity, the outcomes of which were manifestations of adaptation to the college environment including: 1. More enthusiasm for studying at the school and doing academic tasks; 2. More likely to assimilate campus life meaningfully; and 3. More self-perceived personal satisfaction with one’s ability to meet the educational demands at college.

Two key findings suggested that college adaptation might have resulted from the circular causality feedback loops of intention, meaning, and perception. It could be argued that cognitive neurodynamic process may be a significant predictor of college adaptation. Consequently, students’ motivation, meaningful experiences, and perception of themselves as efficacious and satisfied with the outcomes of their academic performance may be embedded and best expressed in the cognitive process of circular causality (Derakhshanrad et al., 2017a).

The findings of this study can be best supported by Freeman’s description of the cognitive neurodynamic process of circular causality phenomenon as a process “in which each perception concomitantly is the outcome of a preceding (intentional) action and the condition for a following (intentional) action.” (Freeman, 1999, p. 148). Just as intentional actions and meaningful experiences build perceptual concepts, so do new perceptions lead the individuals to find new intentions and meanings, which ultimately alter self-perception (Freeman, 2003). Therefore, an argument in favor of supporting the hypothesis of linkage between the cognitive neurodynamic process and students’ adaptation to college would be that adapting and assimilating to the specific condition of the environment can result from effective neurodynamic interactions among intention, meaning, and perception, by which goal-directed behaviors are constructed, implemented, and appreciated by the performer (Freeman, 2000b). In other words, the cognitive neurodynamic process of intention-meaning-perception constructs knowledge about the world, so that it acts as a bridge between the brain and the outside world (Freeman & Burns, 1996).

Few empirical studies about the IMP model make it difficult to relate and evaluate our findings in the light of previous research. However, these findings of the current study are consistent with two earlier similar studies that examined the applicability of the IMP model in studying the academic adaptation construct. Yet, our research has dissimilar methodology and study population to those earlier ones. In the first single-case research method study by Casillas, Davis, Loukas, and Schumacher (2008), it was found that the new primary school’s various educational contexts such as on the school bus, during the initial morning time in the classroom, on the playground, in center activities, and in the cafeteria caused trouble for a new student, resulting the boy having difficulty adjusting effectively and smoothly to various educational demands.

Provided with school-based support services, the child’s circular causality cognitive process was changed, so that he felt he had purpose in the school. Accordingly, he began to assimilate meaningful experiences through his academic performance, such that he built a perception of himself as a student who was capable of learning. In another single-case study by Davis (2009), she emphasized the difficulties of transition to new high school environment that could entail significant challenges for a 13-year-old girl with high-functioning autism. Confronted with increased cognitive demands at school, the girl with special needs had to regulate her cognitive process of circular causality to be able to adapt to school. Thus, the girl did not begin to adapt to her educational setting, until she found new intentions, meanings, and perceptions to tackle the educational issues, as documented by her teacher and rehabilitation staff. For instance, replacing paper-based written assignments with an electronic note taker decreased her anxiety and poor academic habits through improving her perceived self-efficacy, as a result of experiencing a successful and meaningful school experience. On this basis, it may be inferred that institutions that provide academic, social, and personal support services could contribute to facilitate students’ adaptation to college (Consolvo, 2002), possibly through affecting their cognitive process of circular causality.

We suggest that the cognitive process of circular causality merits consideration to understand how college adaptation can be shaped effectively and successfully. According to findings of this study, the cognitive process of circular causality may have enabled adaptation to campus life. The same happened when a sample of patients with strokes started to make changes to adapt to their strokes and it was qualitatively shown that circular causality feedback loops among their intentions, meanings, and perceptions occurred (Derakhshanrad, Piven, & Zeynalzadeh, 2017a). Likewise, the cognitive process of circular causality has been acknowledged to contribute in the adaption of a previously aggressive male client with the history of severely aggressive behavior (Piven & Derakhshanrad, 2017) and two dissimilar female clients with strokes (Derakhshanrad, Piven, & Zeynalzadeh, 2017b).

This study was limited by the use of a convenience sampling method. It should be noted that this causal-comparative study has been primarily concerned with comparing the college adaptation manifestations between students with diverse cognitive neurodynamic processes, as underpinned by the IMP model. Thus, we were unable to determine the responses to some other hypotheses; for example, to name a few: the more one had meaningful experiences, the more he or she perceived himself or herself to be efficacious and satisfied; the more one perceived himself or herself to be effective, the more he or she intended to keep on trying. In addition, research from this study was the first step for our desire to develop a neuro-occupational assessment tool. Standardization research of a tool is yet to come, as we inch toward this long-term goal, to develop a validated tool that can be used in clinical rehabilitation settings with clients. In fact, the BQCNPS used in this study introduced a short inquiry that needs to encompass more questions, so that standardization research using correlational studies to test the concurrent relation between this test and other validated questionnaires can be continued.

We strongly urge that researchers conduct vigorous empirical studies to test whether above mentioned hypotheses are true. In addition, it is recommended that researchers conduct further studies to test whether results would be the same, if they look at students from a broader classification more representative of the students on campus, i.e. postgraduate versus undergraduate students, students taking foreign languages, overseas students, students in engineering, music, mathematics, statistician majors, education students, computer science, etc. Complementary studies are important to further investigate the correlation between academic performance and IMP.

In summary, the results of this study showed that 187 students at an Iranian college may have experienced the cognitive neurodynamic process referred to cognitive process of circular causality. BQCNPS developed for this study has practical potential for further examination and expansion, to pinpoint the cognitive neurodynamic processing capacity. Therefore, it is advisable for faculty and mental health counselors and other authorities who work on campuses to pay more attention to the students’ cognitive neurodynamical circular causality process of intention, meaning and perception, to identify those who may require counseling to improve their adaptation to the college context.

## Ethical Considerations

### Compliance with ethical guideline

This research was approved by Research Ethics Committee of Shiraz University of Medical Sciences, Iran. Informed consent was obtained from all participants. The instruction to students and administration of the research materials were done in classrooms. They were also provided with enough time to complete the research instruments.
